# The crisis of scientific paradigm in modern psychiatry and a look to the future

**DOI:** 10.1002/pcn5.70132

**Published:** 2025-06-08

**Authors:** Santiago A. Levín

**Affiliations:** ^1^ Universidad Nacional Arturo Jauretche Florencio Varela Argentina; ^2^ High Complexity Network Hospital “El Cruce,” Florencio Varela Argentina

**Keywords:** psychiatry, epistemology, paradigm crisis

## Abstract

Contemporary psychiatry is going through times of crisis. In this article, I will focus on the epistemological and historical aspect of the phenomenon. Three authors are the main references guiding the argumentation I offer in this text. They are Thomas Kuhn, Georges Lanteri‐Laura and Juan Carlos Stagnaro. The text is intended as an introduction to the epistemological analysis of contemporary psychiatry, but it also proposes to look a little ahead in order to attempt a modest reflection on the future of the specialty.

## INTRODUCTION

Three authors (Thomas Kuhn, Georges Lanteri‐Laura, and Juan Carlos Stagnaro) before mentioned were specially chosen for this reflection. Thomas Kuhn, because he was one of the first authors of the twentieth century to propose the intimate connection between the production of knowledge and the historical period in which it is constructed. His book *The Structure of Scientific Revolutions* ^1^ was a fundamental contribution to the epistemology of the second half of the last century. Georges Lanteri‐Laura, author of *Essay on the Paradigms of Modern Psychiatry*,^2^ proposes a periodization of the history of the discipline, a contribution we will use for our analysis. His proposal is extremely fruitful and at the same time of high practical utility to understand the current state of the discipline. It is Lanteri‐Laura himself who puts forward the notion of “crisis of scientific paradigm” in contemporary psychiatry, a concept that we will develop later. Juan Carlos Stagnaro, an Argentine psychiatrist and academic, is the author of a work of huge originality and very connected to the aforementioned Lanteri‐Laura,^3^ in which he outlines what he understands as a new paradigmatic proposal for psychiatry, which emerged in the 1990s, coming from the northern hemisphere. In turn, this paradigmatic proposal was explored 20 years later in my own doctoral research (carried out at the University of Buenos Aires between 2014 and 2016).*

At this point, it is important to take into account that history itself as a discipline has undergone profound changes since the second half of the twentieth century. Different currents coexist with the still healthy traditional historiography,^5^, ^6^ which is determined to show the achievements of early psychiatry by describing its pioneers in a heroic key. This historical approach, of positivist origin, had the scientific and social legitimization of psychiatry and its professionals as its fundamental goal.

But in the 1960s, a critical historiography seeking to expand and revise the traditional approach emerged. History opens the doors to the social sciences (anthropology, sociology, etc.) and the so‐called externalist approaches begin to appear. Here it is worth mentioning, albeit very briefly, Michel Foucault and his *History of Madness in the Classical Age*,^7^ Lanteri‐Laura himself,^8^ the so‐called Cambridge School with Berrios and Porter at the head,^9^, ^10^, ^11^ to the sociology of science,^12^, ^13^, ^14^ to mention just a few names. It is very important to point out the fundamental coincidence between all these new currents, despite the controversies and differences: historical knowledge is possible, that is, recognizing the narrative dimension of historical writing does not imply denying its condition of true knowledge, built on the basis of tests and controls.

When turning to the history of psychiatry, our main focus of interest is to unravel the mechanisms by which things are today the way they are. It is interesting to construct a “history of the present,”^15^ which enables us to explain our daily practice as psychiatrists and at the same time the theoretical framework on which we base our knowledge.

That is why we turn to the three authors mentioned from the beginning. Thomas Kuhn's proposal allows us to analyze scientific knowledge from a non‐linear perspective and framed in a historical and social context that in a certain way determines it and at the same time explains it. Lanteri‐Laura's contribution, based on Kuhn's concept of the scientific paradigm, illuminates the history of psychiatry with a new light and subdivides it into differentiated stages (paradigms), ending up in the aforementioned epistemological crisis from the 1980s onwards. Starting from Lanteri‐Laura's proposal and his concept of paradigm crisis, which Stagnaro knew long before its publication, the Argentine psychiatrist makes a remarkable diagnosis: the proximity of a new paradigmatic proposal.

I will make an introduction to the three abovementioned proposals, and then refer to the prolonged epistemological crisis in which our discipline is immersed and, finally, offer a brief personal vision of what I consider should be the future of psychiatry—as a university professor and trainer of psychiatrists I would like to emphasize that a good diagnosis of the present and a minimum idea of the future are essential when designing a training program adjusted to the clinical realities, the historical moment and the social and political context in which the profession will be carried out.

## KUHN AND SCIENTIFIC PARADIGMS

In an article published in 2010, I presented a rather extensive discussion on the scope of the concept of epistemology, framed within philosophy.^16^ For this reason, I will not expand on it herein, instead I will refer the interested reader to this article. It is enough to say that if epistemology is a reflection on scientific knowledge, its birth should obviously take place later. And indeed, if science as we know it, begins in the 16th and 17th centuries, epistemology does not do so until the beginning of the 20th century. “It is no coincidence that precisely at the same time that the crisis of modern science began, reflection on it also began, that is, epistemology.”^17^ It is important to clarify, for the sake of transparency, that I am using a concept of epistemology primarily grounded in the French and continental tradition, whereas in the Anglo–American tradition, the concept tends to cover a broader scope.

There are currents within epistemology that can be globally grouped into two: *internalist* and *externalist*.^18^ The internalist position supports a rather closed definition of science, which emphasizes its internal structure, so it can often fall into that form of reductionism that we call *scientism*. The externalist position, which emerged later (as from the 1960s onwards), situates science as a socially and historically determined discourse and relativizes the notion of scientific truth as immutable and essential. Thomas Kuhn is one of the initiators of this second current.

In 1962, the publication of *The Structure of Scientific Revolutions* points out a real change in the understanding of scientific knowledge. Thus, the historical dimension appears in epistemology, the conception of the scientist situated in an epoch—*their* historical epoch—in a context which determines him. Science thus conceived ceases to be cumulative and universal, governed by indisputable logical–mathematical rules and procedures, and becomes a social production that emerges from its own historical foundation.

Kuhn postulates that science does not “advance” straight forward but evolves in a discontinuous way, presenting crises and reformulations separated by periods of greater stability and consensus. Central to Kuhn's proposal is the notion of *the scientific paradigm*, understood as the set of ideas that provide problems and solutions to a given scientific community over a certain period of time. He calls periods of *normal science* those periods of stability of the scientific paradigm. The paradigm provides the limits of the knowable, defines the limits of the graspable: what is not foreseen by the paradigm is not perceived, clearly an interesting proposal, far from the positivist position. With the passing of time, anomalies begin to appear in the paradigm, gradually weakening it until a *crisis of the scientific paradigm* occurs. A new paradigm comes to replace the previous one. Kuhn postulates that paradigms are incommensurable with each other, that is, they are not comparable point by point as they constitute substantially different versions of reality.

Despite the criticisms Kuhn's proposal aroused (some of which were answered by its own author in later works), its importance for the understanding of science as a historical–social product is indisputable. In the field of psychiatry, Georges Lanteri‐Laura uses the framework proposed by Kuhn to critically analyze the birth and development of our specialty. This will be commented on hereafter.

## LANTERI‐LAURA AND THE PARADIGMS OF MODERN PSYCHIATRY

It was in 1997, at a congress of the Association of Argentinean Psychiatrists (APSA) when Lanteri‐Laura presented his conception of paradigms in psychiatry for the first time. Two years later he published his book *Essay on the Paradigms of Modern Psychiatry*. It is a remarkable work, which aimed to leave a deep mark on the path of understanding the bases on which our specialty is based. Lanteri‐Laura dedicates the second chapter of his book to substantiating the option for the Kuhnian thought as a basic reference. Psychiatry is not a science like astronomy or physics are, so it makes no sense to transpose Kuhn's theory to the letter to make a history of psychiatry. The author will state that it is a matter of making practical use of the Kuhnian proposal with some modifications:

We could conceive the role of the paradigm in psychiatry as the one that unifies for a longer or shorter period of time a whole series of theoretical and practical representations which accommodate to each other or else, exclude each other, as long as that paradigm functions effectively, in an analogous way to what T. Kuhn called normal science.^19^

Another adaptation that Lanteri‐Laura makes of Kuhn's proposal is the following: Kuhn maintained that paradigms are incommensurable with each other and therefore no traces of the old ones are found in the new ones; Lanteri‐Laura affirms that in the history of psychiatry, the old paradigm always leaves traces, splinters, in the one coming after it. While there is a break in continuity, which justifies a periodization, there is also a certain persistence of the previous paradigm in which it continues. This is particularly important in Lanteri‐Laura's proposal, and we will get back to it because it enables us to explain the trace of old concepts in contemporary disciplinary thought.

There is a good deal of consensus in placing the beginning of psychiatry as a medical specialty in Paris in 1789 (the year of the French Revolution) when Philippe Pinel was appointed medical director of the Hospice of Bicêtre.† However, recent historical evidence supports the hypothesis that these beginnings could have been somewhat earlier and not in France but in northern Italy. These findings give visibility to the previously unknown figure of Vincenzo Chiarugi.^20^, ^21^, ^22^ The fact may be more or less important, but it serves to underline the extent to which official history is an instrument of the discourse of the powerful, and during the late 18th century, Paris was the center of the world.

We will mention below the three paradigms of psychiatry proposed by Lanteri‐Laura in 1999, describing each of them briefly, finally leading to the crisis of the scientific paradigm in which, according to this author, our discipline would be placed as from the 1980s to the present.

The *paradigm of mental alienation* begins in 1793 when the Paris Commune appoints Philippe Pinel as medical director at the Hospice of Bicêtre. During this first paradigm, the existence of only one disease is postulated: alienation. “Madness” is understood as a mental pathology, and therefore belongs to medicine. The “foolish” become sick, and can be treated as such (instead of being locked up, or arrested as criminals). Psychiatry is alienism, and the first psychiatrist is the so‐called alienist.

This first paradigm conceives mental illness as a single disease, which has at least three important consequences: (1) mental medicine distances itself from the rest of medicine, renewed by the School of Paris, which maintained the existence of various differentiated entities; (2) this uniqueness is also transferred to institutions and treatment (i.e., all the alienated go to the asylum, all the alienated receive *moral treatment*); and (3) this conception reduces the problem to a binary simplification (i.e., to be crazy/mentally insane or not to be crazy/mentally insane, a reductionism that has not yet been overcome in our days).

The second paradigm was called *the paradigm of mental illness* by Lanteri‐Laura. It begins in 1854, when J‐P. Falret publishes his famous article “On the Non‐Existence of Monomania.”^23^ Falret this way broke with his teacher Esquirol and established that mental pathology should resign to the notion of uniqueness and admit that its field was occupied by several mental illnesses, as well as that of general medicine. A large number of European psychiatrists followed this conception, which reached its peak with Emil Kraepelin between 1890 and 1910. Needless to say, Kraepelin played a central role in the consolidation of the second paradigm. Morbid entities begin to multiply until the paradigm ends up in a crisis again giving way to the need to return to a certain reunification. Lanteri‐Laura spots two problems emerging from this second paradigm: (1) the growing, almost unstoppable clinical plurality, and (2) this diversity reveals that limits in psychiatry are more diffuse than clear and that its content is heterogeneous, a problem that persists to this day.

The *paradigm of psychopathological structures* began in 1926, when the Swiss psychiatrist Eugen Bleuler presented his conception of schizophrenia at the psychiatry congress in Lausanne, Switzerland. Psychoanalysis, phenomenology, the theory of form and the globalist neurology of Kurt Goldstein enter the scene. The novel conception of a psychopathological level transcending the clinic led psychiatrists in the first half of the twentieth century to no longer abide to the exhaustive list of mental illnesses. The problem in this third paradigm may be synthesized in being neurotic or being psychotic, enriched by Henri Ey's conception of madness as a pathology of freedom.^24^, ^25^

This third paradigm came to an end with the death of the French master Henri Ey in 1979, a full representative of this historical period and the last holistic psychiatrist who tried to unify a general theory about human beings and their different ways of getting sick both of the body and the mind: organo‐dynamism.

## CRISIS OF SCIENTIFIC PARADIGM

We agree with Lanteri‐Laura on the characterization of the present of our specialty as a long period of crisis of scientific paradigm. This means that there is currently no definition about what psychiatry is today that is accepted by the entire community of colleagues; what its scope is, its limits with other areas of knowledge, that is, its specificity; what would be an acceptable classification of nosological entities and what would be the most appropriate therapy to address each of them.

Lanteri‐Laura says that conceptions have been added to those already established, setting up a panorama in which psychopathological references multiply with some dominating more than others, at least initially. At the same time, the distances separating daily clinical and therapeutic activity from the elaboration of theories has increased significantly and “we very much miss a *theory of practice* capable of explaining these practices in a reflexive way” (Lanteri‐Laura, 1999).^26^ And he goes on to say: “Now, if such considerations clearly show that the third paradigm cannot fulfill its role, they do not provide us with any consistent element to imagine what the fourth paradigm will be like.”

And this is where the 1996 Stagnaro comes in. Despite having been published in 1999, it is very likely that the writing of several of the chapters that make up the book *Essay on the Paradigms of Modern Psychiatry* began much earlier. Likewise, the friendship and exchange between Lanteri‐Laura and Stagnaro, who lived in France for several years. The work read by Stagnaro in 1996 at the tenth Conference of the Association of Psychiatrists of Córdoba, Argentina, already includes the content of Lanteri‐Laura's book, which was to be published 3 years later. That is why Stagnaro speaks of a “candidate for the fourth paradigm,” taking for granted the still unpublished production of his colleague and friend.

## A CANDIDATE FOR THE FOURTH PARADIGM

In general terms and by definition, it is not possible to “see” outside the paradigm in which the observer is immersed. If the paradigm in which the observer is immersed is in full crisis, and if the observer has the necessary conceptual tools, this could present an exception to the rule. And this is what happened with Stagnaro's work in 1996. It is worth highlighting the curious fact that the work we mention, *Critical Study of the Current Paradigmatic Crisis in Psychiatry*, was never formally published—and yet it is the most quoted text by its author.

Stagnaro locates the fourth paradigm candidate as coming from the Anglo‐Saxon world, in particular from some universities and research centers in the northern hemisphere—later I will introduce the notion of *invisible colleges*, indispensable to understand the source of Stagnaro's interpretation.

The 1990s was the “decade of the brain.” It was a period of sharpening cultural globalization after the fall of the Berlin Wall with a clear increase in the world hegemony of the United States of America. In the mid‐1990s, by analyzing papers, congress programs, books of the specialty, and the recently published *Diagnostic and Statistical Manual of Mental Disorders* (DSM IV),^27^ Stagnaro glimpses a candidate for a fourth paradigm. He conceives it as supported by three interconnected and interdependent statements:

1. It is possible to identify mental disorders (syndromes) in an objective way (this is stated in the Introduction to the DSM IV).
2. It is possible to draw a bi‐univocal correlation between each disorder thus described and its brain pathophysiology (this is the central “promise” of the so‐called decade of the brain).
3. If both previous statements are correct, then it is possible to establish that this pathophysiology will be adequately corrected by pharmacological therapy combined together with psychotherapy originated in the theory of learning (basically cognitive behavioral therapy).

Looking at it almost three decades later, it is very clear that this was the program proposed by American psychiatry at the end of the last century. Likewise, seen from the year 2025, Stagnaro's work was a transcendental contribution to the understanding of the historical course taking Western psychiatry to lead a proposal of this type, which we have characterized as biological reductionist or bioreductionist, and which has never reached (as we will see later) the status of a consolidated paradigm.

We consider it essential to introduce here a passage by Germán Berrios, whose perspective is key to properly framing this issue:

Clinical psychiatry is not doing well, and it is legitimate to hypothesize that this failure is due to the fact that all of its ideas and data come from biological psychiatry. Bringing to light the reasons for which this situation has come to light posts an urgent task for the epistemology of psychiatry. We need to understand why and how the current language and objects of psychiatry were constructed. This work will require a joint effort of historians and philosophers of psychiatry.^11^

Once Lanteri‐Laura's third paradigm got into crisis, clearly originated in the European continent, this paradigmatic proposal emerged from the centers of thought of the United States of North America. The decade of the brain, the Human Genome Project, evidence‐based medicine, the strong presence of the pharmaceutical industry, and the flourishing of neurosciences are some of the elements that characterize the context of its emergence at the end of last century.

At this point, the concept of “invisible schools” becomes necessary. Gerald Klerman,^28^ an American psychiatrist, director of the Substance Abuse and Mental Health Services Administration (SAMHSA) of the United States between 1977 and 1980, was the first to speak of the “invisible school of the DSM.” He was referring to the DSM working group and other figures in the field of mental health in his country, who coincided with the nosographic opinions of Robert Spitzer (a central figure in 20th‐century American nosology), who led the DSM III working group in 1980.

The term “invisible college” started to be used in the 17th century by Elias Ashmole, an English politician and soldier and one of the founders of the Royal Society of London as well as the Freemasonry, the latter linked to secrecy and stealth.^29^ An invisible college is a group of specialists who, without being organically united, coincide in certain ideas and converge in publications and academic activities.

For Klerman, the characteristics of the invisible college of the DSM are the following^30^: (1) psychiatry is a branch of medicine and must be founded on science; (2) there is a clear limit between the normal and the pathological; (3) there are different mental illnesses; and (4) the biological dimension of these diseases is mostly important.

To sum up, Stagnaro “reads” (diagnoses) a paradigm proposal during the mid‐1990s. He does so by analyzing the invisible college production of biological reductionism in psychiatry, and by carefully observing every move on the chessboard of the specialty (publications, symposia, industry sales pitches, general health market trends, scientific congress programs, academic productions, modifications in the curricula of medical careers and specialization in psychiatry, prescription fashions, the syllabus of refreshing courses, etc.).

Knowing that it is impossible to address every detail in this article, it is important to mention the influence of the neo‐Kraepelinian group from Washington University in St. Louis, led by Eli Robins and Samuel Guze, on Spitzer's nosological views. We believe this influence—along with the Fighner criteria—was decisive in the conception and development of the DSM‐III, a milestone in the neo‐Kraepelinian movement.^31^, ^32^

Following the Kuhn and Lanteri‐Laura line, Stagnaro places his analysis within the framework of a particular periodization formalizing, with remarkable success, what he considers a proposal for a new paradigm for psychiatry. This proposal does not bear the signature of any psychiatrist but has its origin in the aforementioned invisible college, which we have come to call “invisible college of biological reductionism in psychiatry,” or, like Klerman, “invisible college of the DSM.”

In order to make a phenomenon visible, it is necessary to have an adequate conceptual structure. Revealing a phenomenon not yet seen implies a conceptual renewal. The new concepts illuminate, make visible and enable us to go on researching and continue to gather knowledge. Perhaps this is the most transcendent mission of science.

## THE CANDIDATE FOR THE FOURTH PARADIGM UNDER SCRUTINY

Between 2014 and 2016, I carried out my doctoral research^33^ under Juan Carlos Stagnaro's tutorship. The aim of my work was to find academic support for diagnosis,^3^ and at the same time to investigate what had been the fate of the candidate for the fourth paradigm of psychiatry.

My initial hypotheses were threefold: (1) towards the end of the 20th century, there was a proposed paradigm for psychiatry characterized by its bioreductionist bias; (2) despite not having reached the status of an established paradigm, it had a decisive weight in Western psychiatry; and (3) the paradigmatic proposal then went into decline due to problems in its internal coherence and explanatory power, further deepening the paradigm crisis that began in 1980.

In order to verify and contrast these three hypotheses, I designed a methodology which I will not develop here for space reasons. The same can be consulted by resorting to the thesis itself^33^ or to the book published below with the results of the research.^4^ But as methodology is one of the keys in doctoral research, its fine‐tuning required several adjustments. Very succinctly, a deep web search strategy was designed from specially selected *descriptors*. As a result, we obtained a total of 846 *papers* taking a time span from 1975 to 2014 (that is, from shortly before the decline of the third paradigm to the exact date of the start of the research).

In the aforementioned period, it is evident that the number of articles “attracted” by the selected descriptors (“nosography,” “neurobiology of mental disorders,” “neuroimaging,” “neurophysiology,” “genetics,” “psychopharmacology,” “cognitive behavioral therapy”) increased progressively as the year of publication increased. This makes it evident that in the period under consideration, there was a clear shift from a theoretical framework based on psychoanalysis/psychopathology (Lanteri‐Laura's third paradigm) to a perspective based on the biological aspect of the mental.

Our hypotheses could be demonstrated using the aforementioned methodology and its qualitative quantitative analysis. What was a hypothesis in the mid‐1990s, an idea‐diagnosis based on the observation and analysis of the text and context of Western psychiatry at the end of the century, is today a concrete object of study in the epistemology and history of psychiatry. We have been able to establish that there was indeed the intention to construct a non‐theoretical nosography, that is, a classification in its pure state, without “contamination” that could distort its essence.

Stagnaro distinguished an approaching comet in a starry sky. A few years later, we could see it unfolding in the sky of psychiatry, acquiring an enormous weight, proportional to the political and economic power of the countries in whose academic centers it originated. Everything seems to indicate that although it changed the “environmental conditions” of psychiatry for 30 years, the comet is passing by. The figure of the “invisible schools,” both a metaphor and a concrete object of study, is fundamental to understanding this phenomenon in a complete way.

## CRACKS THROUGH WHICH THE PARADIGMATIC PROPOSAL FAILS

In the doctoral research, three weak points (anomalies) of the bioreductionist proposal that we have been analyzing were explored in depth, and the three (which we have decided to call “cracks,” such as the cracks in the retaining wall of a hydroelectric dam) refer to pharmacology: the efficacy, resistance and methodology of the Controlled Clinical Trial.‡ There are only three among several, but they are the ones I choose to illustrate the phenomena from within the bioreductionist proposal. How can it be that what is observed in experimentation does not happen in our clinic? Are we, the clinicians, the ones who are wrong, or is there something wrong as the texts of the specialty foresee and as is repeated in congresses over and over again?

We can find several anomalies looking at the paradigm from the inside. For example, there are a significant number of patients who do not respond to pharmacotherapy. On the other hand, another group cannot manage to recover the previous state—that is, a certain response is obtained but not a complete recovery—. There are indeed clinical research designs that reflect little of the real patient (the one who consults us daily) and whose conclusions are, therefore, of quistionable application. The prescription of antidepressant drugs is significantly increasing worldwide, and there is no decrease in the health problem of depression or the suicide rate.

Since the end of the last century, we have been observing announcements about the “discovery” of the gene responsible for some mental disorders, an inadmissible reductionism that only generates confusion and disappointment in a public eager for answers. In this sense, it is advisable to compare the position of authors such as Althoff and Waterman,^34^ who advocate a psychiatry based solely on genetics and neuroscience, with that of geneticists with a broader conception of the human being, such as Víctor Penchaszadeh,^35^ or philosophers such as Natalie Banner, author of an extraordinary article entitled “Mental disorders are not brain disorders.”^36^ I would also add Nancy Andreasen^37^ to this short list, a renowned psychiatrist and neuroscientist who advocates to focus all interest exclusively on the brain (“What we call ‘mind’ is the expression of the brain's activity”), while Banner states: “The view that everything mental is supported by the brain is wrong, because the level at which we identify mental disorders is, and should be, the person, not the brain.”

The discussion between biological reductionist positions and more comprehensive conceptions (which we call anthropological) is a complex, fascinating and still unfolding discussion. We could say that both positions carry a part of truth, that the biological reductionist position continues to predominate (and by a large margin) and that there are also non‐biological reductionisms, such as psychological (everything is a product of Oedipus) or sociological (there is no madness but only the social label to everything different).

When we state that the candidate for the fourth paradigm failed in its attempt to impose itself as a solid paradigm accepted by the entire scientific community, we do not mean that biological reductionism has lost ground. Definitely not. We only state that this proposal diagnosed by Stagnaro in 1996 and emerging from the academic centers of the northern hemisphere as a product of the action of the invisible college of the DSM (initiated in 1980 with the publication of DSM III) did not manage to stabilize itself as an established paradigm. A non‐theoretical and objective classification was not maintained (non‐theoreticity does not exist); the bi‐univocal correlation between each disorder and its brain pathophysiology has not been found, and the expected success was not achieved with psychopharmacological therapy. The candidate for the fourth paradigm depended for its consolidation on the realization of promises that were left unfulfilled.

But the reductionist approach remains in the most generalized way of thinking about mental pathology, in congress programs, in specialized publications and, above all, in undergraduate curricula (and this with very few exceptions). The history behind this reality is long and complex and exceeds the framework of this writing, but it is essential to mention the effect of the positivist approach of the mid‐19th century, the Flexner report of 1910, the political and social changes in the West as a result of the two world wars, the progressive co‐modification of health and the growing and determining influence of the interests of the powerful pharmaceutical industry. Each of these elements deserves its own development, but we cannot ignore the fact that certain extra‐scientific interests have greatly influenced (and continue to do so) so that mental phenomena are thought of in a certain way and not in a different, more comprehensive, less reductionist way. The texts of Philippe Pgnarre^38^ and Marcia Angell,^39^, ^40^ among others, are indispensable for an in‐depth understanding of this phenomenon. Regarding the reductionist approach as a whole, it is crucial, in attempting to understand it, to distinguish between the influence of positivism—a “way of thinking” rooted in a specific philosophical tradition—and other “external” factors, such as economic interests (e.g., the pharmaceutical industry) and various social changes that occurred during the 20th century.

It is necessary to mention, even briefly, the numerous critiques and debates that surrounded the launch of the DSM‐5.^41^ “Diagnostic inflation,” the influence of Big Pharma, and the increasingly blurred boundaries between normality and pathology are some of the issues under discussion and—in our view—contribute to the notion of a paradigm crisis.

We have already mentioned Gerald Klerman's publication in 1978. From our point of view, it is a clear example of the intention, on the part of an important and influential group of psychiatrists belonging to the most powerful country in the world, to impose a way of conceiving psychiatry that coincides with Stagnaro's description of a proposal for a scientific paradigm.

This type of conception, based on a descriptive classification, as stated by Gasser and Stigler, “doesn't come without consequences for psychiatry and for general medicine.”^42^ The emphasis placed on pharmacological and epidemiological research as well as the optimization of its conditions (large homogeneous groups, minimum of intervening factors, inter‐judge fidelity, etc.) clearly privileges the hospital domain in the diagnostic and therapeutic approach, at the expense of the individual clinical approach of the clinician (psychiatrist or generalist) who, more intuitive and focused on the link and the context, accepts its incompleteness and its openness to the changes brought about by the evolution of the picture (own translation).

This exposes one of the biggest problems in this type of approach which has become hegemonic: the separation between research and clinic, a phenomenon derived from the success of the DSM's proposal for the invisible college and its reductionist stance. The book just cited is coordinated by the sociologist Alain Ehrenberg and the anthropologist Anne Lovell^43^ and has a more than suggestive title: *Mental Illness in Mutation*. Psychiatry is a *special medicine*, say the compilers in the introduction, because it is situated at the intersection of the medical, the social and the moral. And for this very reason, psychiatry is the subject of constant debates and controversies, “not only concerning the efficacy of therapeutics, research strategies, the etiology of diseases, but philosophical and moral questions concerning the conception of the human subject as well.” Other branches of medicine, such as traumatology or cardiology, do not cross these districts. “Psychiatry is concerned with a particular type of pathology, in which the subject's capacity to evaluate their own suffering is involved.”

## WHERE ARE WE HEADING?

In the background of the current problems in our psychiatry there is a dispute, a tension, between two opposing models: biomedicine, on the one hand, and anthropological medicine, on the other.^44^ On the one hand, a reductionist psychiatry that adapts to the market's need not to waste time on subtleties and particularities; on the other, a comprehensive model with its attention on the *person's level*, following Natalie Banner. It is this second psychiatry that must now take the initiative and engage with the neurosciences, aiming to transform a frequently reductionist perspective into one capable of shaping the future. A perspective that integrates the biological dimension alongside all others to construct a comprehensive understanding of the human being, both in theoretical discourse and clinical practice.

“The natural sciences alone cannot create new categories of ‘mental disorder,’ any more than any somatic imprint can be enough to define a mental disorder. This means that the human sciences have epistemological primacy.”^11^ There is no biological diagnosis in psychiatry. The diagnosis is made by resorting to a technique as old as it is sophisticated: the clinic. A clinical practice that must be informed by—and carried out with—biology, never without it. The main criticism here is directed at reductionism—all forms of reductionism—not at biology itself, which remains an essential component of our being. And when we say “there is no biological diagnosis in psychiatry,” we refer to the absence of primary biological markers for psychiatric disorders—not to secondary psychiatric symptoms of organic origin (e.g., those caused by a brain tumor). In distinguishing between the two, biology and somatic tests and examinations are essential.

As a wrap up, I will share some reflections published together with Daniel Matusevich in an editorial of the year 2022 in the Argentine magazine *Vértex*,^45^ a text we have called “Psychiatry has a future.”

Psychiatry has been the target of criticism since its very origins at the end of the 18th century: for lagging behind scientific‐naturalistic medicine; for the taxonomic excesses of the 19th century; for the asylum system; for the use of electroconvulsive therapy; for the social labeling implicit in diagnosis within an intolerant world; for its lack of a materially grounded etiology; for the multiplicity of competing psychopathological theories; for its collaboration with repressive regimes (e.g., in Nazi Germany, Soviet Russia, and in more familiar contexts as well); for the use—or the refusal—of psychotropic drugs; and for the prolonged duration of many psychotherapies. No other medical specialty has been subjected to such sustained reproach or scrutiny.

In 1978, Henry Ey published *In Defense of Psychiatry*, a work completed just 4 months before his death, in which he tried to respond to the contradictions and criticisms of the time. In this brief text, Ey substantiates the belonging of psychiatry to medical science —“psychiatry is medical or it is not”—and leaves behind as one of the great lessons that the specific object of psychiatry is not the brain, the neuron, the synaptic cleft, the molecule, the consciousness or the unconsciousness but the human being as a whole. If mental illness is above all dehumanization and loss of the self, then everything that dehumanizes concerns the psychiatrist.

Ey helps us understand that, despite all the criticism, there seems to be something irreducible—something he called the *psychopathological fact*. A hard core that resists fragmentation and appropriation by other disciplines or fields of knowledge, transcending nomenclatures and changes of era. Madness exists. There are suicides. There are psychotic decompensations. There are anxiety attacks—in a thousand forms and under a thousand names. There are addictions: drug withdrawal, acute intoxication, cortical failure in the context of organic disorders. There is frank mania, and hypomania—less obvious, yet marked by unstoppable impulsivity and compulsions. There are phobias, some of which remain inaccessible to psychotherapies. There are crises in human relationships, which increasingly lead to violence. There are the cognitive struggles of those who are losing their memory, and there is also the psychic expression of civilizational exhaustion—in an inequitable and ruthless world that, minute by minute, produces suffering that becomes symptom: pain that is inexplicable and unclassifiable within a purely disciplinary taxonomy.

Only 40 years ago, the time when Henri Ey was writing, was epistemologically more stable. On the other hand, both current study texts and papers barely manage to account for the changes in a world in constant transition in which some no longer speak of clinical but of *post‐clinical*. Any practice or knowledge must today inhabit a place of epistemological humbleness. And from such humbleness accompany pain with no other certainty than the centrality of the human bond, taking our theories as a necessary and transitory support. Knowledge that closes in on itself only produces theoretical narcissism, with no possibility of escape either for those holding it or for those who are assisted. Nor should practices close in on themselves: it is no longer disputed today that users and relatives are an indispensable part of the construction of a more horizontal therapeutic project, leaving paternalism behind and enabling a consensual therapeutic design.

A few years ago, Norberto Conti lamented the *de‐philosophization* of contemporary psychiatry, that is, its notorious resignation of reflection on its own foundations and scope.^46^ The reverse path is urgent. A *re‐philosophization* that places us back in the world of those who think and think about themselves, of those who worry about asking themselves on a daily basis about the tools available to the clinic, without supplying any of them as something already given, become doctrine or religion. The question about the foundations—the genealogies, the epistemologies—of our work is not only due to the intellectual restlessness or the practical needs posed by a changing clinic, but also constitutes, above all, an ethical position, more necessary than ever when certainties are scarce.

Having said all of the above, it remains to be added that psychiatry has a future—but only if it finds a way out of the reductionist trap and returns to the path of philosophical reflection, without which the understanding of the human phenomenon is impossible, especially in socially and culturally complex times marked by constant and rapid change. Psychiatry has a future insofar as it seeks to integrate the multiplicity of “islands” that currently compose it—being more an archipelago than a continent. The urgent task is to connect these isolated sectors while simultaneously striving to understand a reality in constant mutation, one that continually reshapes the vital conditions from which new ways of being and existing in the world emerge.

## AUTHOR CONTRIBUTIONS

N/A.

## CONFLICT OF INTEREST STATEMENT

The author declares no conflicts of interest.

## ETHICS APPROVAL STATEMENT

N/A.

## PATEINT CONSENT STATEMENT

N/A.

## CLINICAL TRIAL REGISTRATION

N/A.

## Data Availability

N/A.

## Footnotes

* The title of the thesis was “Critical study of the decline of the bioreductionist paradigm proposal for contemporary psychiatry”, and it was carried out at the Faculty of Medicine of the University of Buenos Aires, with Juan Carlos Stagnaro as director. The thesis was defended in November 2016, receiving an outstanding grade from the jury. Later, this thesis was transformed into a book published by the University of Buenos Aires publishing house.^4^

† We are not talking here about the history of madness, a history that is probably thousands of years old, but about the birth of (Western) psychiatry as a new medical specialty, an event that occurs towards the end of the 18th century. To broaden the panorama, it is recommended to visit the work of Vincenzo Chiarugi (Collection of Classics of Psychiatry, Editorial Pólemos, Buenos Aires).

‡ For a detailed development, I refer the reader to the text of the doctoral thesis and the book published later (Levin, 2016 and Levín, 1019). It would not make sense to expand on this important topic in a short article.
